# Management Strategies for Hypercapnic Respiratory Failure in Chronic Obstructive Pulmonary Disease (COPD): A Case-Based Discussion

**DOI:** 10.7759/cureus.84717

**Published:** 2025-05-24

**Authors:** Bedih Balkan, Ebru Kaya, Gökçe Gokoglu, Ali Osman Balkan, Gülseren Yilmaz

**Affiliations:** 1 Department of Adult Intensive Care, Kanuni Sultan Suleyman Training and Research Hospital, Istanbul, TUR; 2 Department of Intensive Care Unit, Kanuni Sultan Suleyman Training and Research Hospital, İstanbul, TUR; 3 Department of Anesthesiology and Reanimation, University of Health Sciences, Kanuni Sultan Suleyman Training and Research Hospital, Istanbul, TUR; 4 College of Medicine, Bezmialem University, Istanbul, TUR; 5 Department of Anesthesiology and Reanimation, Kanuni Sultan Suleyman Training and Research Hospital, Istanbul, TUR

**Keywords:** high-flow nasal oxygen (hfnc), hypercarbic respiratory failure, icu (intensive care unit), invasive mechanical ventilation (imv), non-invasive mechanical ventilation (nimv)

## Abstract

Hypercarbic respiratory failure due to chronic lung disease is common and presents significant challenges, especially as many patients have multiple comorbidities. Chronic obstructive pulmonary disease (COPD) is one of the leading causes. Patients with COPD and respiratory failure, whether acute or chronic, face worse prognoses. Managing exacerbations of chronic obstructive pulmonary disease (COPD) requires a personalized approach, taking into account the patient's specific clinical profile and comorbid conditions. High-flow nasal cannula (HFNC) therapy has been shown to prevent the need for intubation and may aid in the early extubation process. In patients with COPD who develop hypercapnic respiratory failure while in the intensive care unit (ICU), the decision between high-flow nasal oxygen (HFNO), noninvasive ventilation (NIV), and invasive mechanical ventilation should be made based on the severity of respiratory failure, the patient's overall health status, and individual characteristics. The selection of the appropriate ventilatory support method must also consider factors such as the degree of respiratory distress, blood gas abnormalities, and the presence of other underlying medical conditions that could influence the response to treatment. Each intervention has its indications, benefits, and limitations, and the optimal choice should be tailored to the patient's needs. Noninvasive ventilation (NIMV) has become this population's primary respiratory support method, though comfort issues can hamper patient compliance. High-flow nasal oxygen (HFNO) devices have emerged as a valuable alternative. This study presents cases illustrating the application of conventional NIMV, HFNO, and invasive ventilation in treating hypercarbic respiratory failure.

Written consent was obtained from the patient's relatives for all procedures to be performed on all three patients after admission to the intensive care unit.

## Introduction

Acute exacerbations of chronic obstructive pulmonary disease (COPD) account for most COPD-related costs and, when frequent, lead to marked reductions in health-related quality of life [1]. Acute exacerbations of COPD (AECOPD) often lead to hospitalization, accelerated lung function decline, and increased mortality, necessitating pharmacological and respiratory support, including oxygen therapy, a high-flow nasal cannula (HFNC), non-invasive mechanical ventilation (NIMV), and invasive mechanical ventilation (IMV) [2]. NIMV is the first-line intervention for AECOPD with hypercapnic respiratory failure, as it reduces intubation rates and mortality [3]. However, patient intolerance due to mask discomfort or claustrophobia may limit its efficacy [4]. 

HFNC has emerged as an alternative for patients who cannot tolerate NIMV or have contraindications. It delivers heated, humidified oxygen at high flow rates, providing low-level positive airway pressure, reducing anatomical dead space, and improving secretion clearance [5-6]. In cases of severe respiratory failure refractory to NIMV or HFNC, IMV becomes necessary, though it carries risks such as ventilator-associated pneumonia and prolonged weaning [7]. Managing COPD exacerbations requires a tailored approach based on the patient's clinical profile and comorbidities. HFNC can prevent intubation and facilitate early extubation. For patients with chronic obstructive pulmonary disease (COPD) experiencing hypercapnic respiratory failure in the intensive care unit (ICU), the choice between high-flow nasal oxygen (HFNO), noninvasive ventilation (NIV), and invasive ventilation depends on the severity of the respiratory failure and patient-specific factors.

This case series highlights the complementary roles of NIMV, HFNC, and IMV in managing hypercapnic respiratory failure due to AECOPD, emphasizing individualized treatment based on clinical severity and patient tolerance. 

## Case presentation

Case 1

A 63-year-old male with COPD, diabetes mellitus, and hypertension presented to the emergency department with shortness of breath. He had a 30-year smoking history and a prior 18-day ICU admission due to COPD exacerbation. He was diagnosed with deep respiratory acidosis and admitted to the ICU. Treatment began with bilevel positive airway pressure (BIPAP) via NIMV and inhalers. Auscultation of the lungs revealed bilateral crackles and computed chest tomography showed pneumonic infiltrates. Given the pneumonia, antibiotic treatment was initiated, and BIPAP treatment continued. Arterial blood gas showed (Table 1) a pH of 7.15, a partial pressure of carbon dioxide (pCO2) of 141, and bicarbonate (HCO3) of 36.1. The patient required intubation after six hours of treatment, and mechanical ventilation was set to pressure-controlled mode (positive end-expiratory pressure [PEEP]: 6, FiO₂: 40%, Frequency: 14, Tidal Volume: 18); equal ventilation in the lungs was observed bilaterally; however, dense secretions were also noted in the intubation tube. Antibiotic therapy was adjusted based on culture results, and sedation was reduced to assess consciousness and spontaneous respiratory effort. The patient was extubated on the eighth day. The arterial blood gas (ABG) obtained that day showed a pH of 7.36, pCO2 of 70, and HCO3 of 33.9. The patient was transitioned to high-flow nasal oxygen support (50 L/min, FiO₂: 40%, 37°C). *NIMV was used 4x1 hours per day for 48 hours, and HFNC was used for 20 hours per day. It started with high currents and then switched to low currents, and then nasal oxygen was used.* The patient was discharged on the 12th day of treatment. On the day of discharge, his ABG showed a pH of 7.38, PCO₂ of 57.4, and HCO₃ of 31.1 (Table 1). In case 1, chest radiographs showing significant improvement at admission were compared with chest radiographs showing significant improvement at discharge. By using a combination of NIMV, HFNC, and IMV, the patients' hypercarbic respiratory failure was effectively managed.

**Table 1:** Ventilatory management and blood gas analysis in three COPD patients with hypercapnic respiratory failure. COPD: Chronic obstructive pulmonary disease

Variable	Case 1	Case 2	Case 3
Age (years)	63	60	74
Gender	Male	Male	Female
Primary Diagnosis	Acute exacerbation of COPD	COPD	Congestive heart failure, Asthma
Comorbidities	Diabetes mellitus, Hypertension, COPD	Tuberculosis (2004), Scoliosis	Hypertension, Type 2 Diabetes Mellitus, Hyperlipidemia
Admission Symptoms	Shortness of breath	Sudden shortness of breath	Sudden shortness of breath
Invasive Mechanical Ventilation	Yes, with lung-protective strategies	Yes, with lung-protective strategies (until day 8)	No
BIPAP	Yes	Yes	Yes
High-Flow Nasal Oxygen (HFNO)	After extubation, up to 40 L/min	Up to 60 L/min	No
Outcome	Discharged on day 8	Deceased on day 20	Discharged on day 12
ABG Before Intubation			
– pH (7.35–7.45)	7.15	7.23	7.18
– pCO₂ (35-45mmHg)	141	91	130
– pO₂ (60-100mmHg)	65	150	60
– HCO₃⁻ (22-26mEq/L)	35.5	31	38
– Base Excess (+3,-3mmol/L)	19	9.6	20
ABG After Intubation			
– pH	7.34	7.34	7.30
– pCO₂ (mmHg)	78	70	85
– pO₂ (mmHg)	95	114	90
– HCO₃⁻ (mEq/L)	35.3	33.1	36
– Base Excess (mmol/L)	12	11.1	15
ABG After Extubation			
– pH	7.36	7.44	7.40
– pCO₂ (mmHg)	70	68.8	65
– pO₂ (mmHg)	95	74.1	85
– HCO₃⁻ (mEq/L)	33.9	44.1	35
– Base Excess (mmol/L)	13.5	21	14

In case 1, the pH was 7.15 before treatment and increased to 7.38 after treatment. Similarly, pCO2 values decreased from 141 mmHg to 57.4 mmHg." In case 1, the patient's respiratory acidosis (pH 7.15, PCO₂ 141 mmHg) and heavy secretions led to a decision to intubate after 6 hours. *The patient first received non-invasive ventilation, but due to worsening respiratory failure (evidenced by respiratory acidosis), they required intubation and invasive ventilation. After recovery and extubation, the medical team used a combination of high-flow oxygen and non-invasive ventilation to help the patient fully wean off mechanical support and regain normal breathing.*

Case 2

A 60-year-old male patient with known diagnoses of COPD, scoliosis, and a history of tuberculosis in 2004 was admitted to the emergency department with complaints of dyspnea and tachypnea. BIPAP treatment was started in the emergency room, but as the patient's condition worsened, he was transferred to the intensive care unit. The patient received high-flow nasal oxygen (HFNO), with settings recorded at the start of treatment (flow rate: 50 L/min, FiO2: 40%, and median temperature: 37°C). Additionally, non-invasive mechanical ventilation (NIMV) was used, and the chest X-ray showed significant.

On the 10th day of hospitalization, the patient's Glasgow Coma Scale (GCS) was measured at 7, prompting intubation due to deep respiratory acidosis, as reflected in the patient's arterial blood gas results (Table 2). After intubation, he was connected to a mechanical ventilator. When arterial blood gas normalized on the 14th day, the patient was extubated and placed under high-flow nasal oxygen support with NIMV treatment. A chest X-ray after extubation showed significant opacities that were not very different from the one at the admission.

However, on the 16th day, the patient exhibited a significant elevation of infection parameters and was treated for septic shock. He experienced desaturation and regression in GCS, leading to re-intubation and monitoring in PRVC mode on the mechanical ventilator. On the 20th day, while receiving high-dose positive inotropic support, the patient suffered a cardiac arrest, and CPR (cardiopulmonary resuscitation) was initiated. Unfortunately, CPR was unsuccessful; the patient was declared dead.

In case 2, the patient's length of stay in the intensive care unit remained 20 days, and complications developed due to septic shock. These findings support the effectiveness of treatment modalities. In case 2, the combination of NIMV (non-invasive mechanical ventilation) and HFNC (high-flow nasal cannula) was initially preferred due to the patient's scoliosis and tuberculosis history. *However, when the patient's condition worsened (likely due to septic shock), treatment was escalated from NIMV to invasive mechanical ventilation.*

Case 3

A 74-year-old female patient with a history of hypertension, type 2 diabetes mellitus, congestive heart failure, asthma, and hyperlipidemia presented to the emergency room with complaints of swelling in her legs for the past 10 days and dyspnea that started on the same day. The patient's blood pressure was 148/88 mmHg, her pulse was 72/min, her oxygen saturation was 90% on room air, and her respiratory rate was 34/min. Additionally, +3 pretibial edema was noted bilaterally. Blood gas analysis revealed respiratory acidosis (Table 1), prompting her to transfer to intensive care. Due to persistent respiratory acidosis and ongoing complaints of dyspnea and tachypnea, the patient was monitored in the intensive care unit for decompensated heart failure management and non-invasive mechanical ventilation (NIMV). She was alert, oriented, and cooperative during hospitalization, with a Glasgow Coma Scale (GCS) score of 15. Her blood pressure was recorded at 135/76 mmHg, pulse at 89/min, oxygen saturation at 92% under 4 L/min of oxygen support, and respiratory rate at 28/min, using accessory muscles in respiration. Examination of the lungs revealed bilateral rhonchi and intermittent rales. Computed chest tomography showed centriacinar ground-glass density with micronodules and a mosaic attenuation pattern in both lungs (echocardiography revealed an ejection fraction of 40%). The patient was started on broad-spectrum antibiotics, inhaler therapy, and diuretics. BIPAP treatment was applied using NIMV. NIMV treatment was tapered intermittently and was discontinued on day 12. The patient was discharged on the 18th day with a recommendation for a follow-up appointment at the pulmonary diseases specialist outpatient clinic. Chest X-ray and arterial blood gas showed significant improvement at the time of discharge (Table 1).

In case 3, *the choice between early intubation and non-invasive mechanical ventilation (NIMV) alone depended on the severity of respiratory acidosis, clinical trajectory, and patient compliance. NIMV sufficed because the patient had milder respiratory acidosis (pH likely >7.25, PaCO₂ <60-65 mmHg) and was cooperative, allowing effective NIMV adjustment.​​​​​​​*

## Discussion

Chronic obstructive pulmonary disease (COPD) is frequently complicated by acute exacerbations characterized by increased dyspnea, often necessitating hospitalization [8]. Among patients presenting with acute hypercapnic respiratory failure and respiratory acidosis, non-invasive mechanical ventilation (NIMV) remains the first-line therapy. NIMV has been shown to reduce the work of breathing, improve gas exchange, shorten hospital stays, decrease the need for endotracheal intubation, and lower mortality rates [9,10]. However, patient intolerance due to discomfort, mask-related issues, or anxiety can limit its effectiveness.

When NIMV fails or is poorly tolerated, clinicians must consider escalation to invasive mechanical ventilation (IMV) or alternative supportive measures. IMV is often associated with poorer outcomes in COPD patients, including increased risk of ventilator-associated complications, prolonged hospitalization, and higher mortality when compared to NIMV [11].

*HFNC in hypercapnic respiratory failure: critical assessment advantages: HFNC improves comfort, reduces dead space, and may lower PaCO₂ by flushing nasopharyngeal CO₂, as shown in respiratory care 2020 [12,13].* HFNC delivers heated, humidified oxygen at high flow rates (up to 60 L/min), which enhances mucociliary clearance, reduces airway inflammation, and maintains epithelial integrity [13]. In addition, HFNC provides a degree of positive end-expiratory pressure (PEEP), which can mitigate intrinsic PEEP (PEEPi), reduce the work of breathing, and assist in carbon dioxide clearance by washing out anatomical dead space [14]. Clinically, HFNC has improved oxygenation, reduced respiratory rate, and enhanced patient comfort. However, its efficacy in severe hypercapnia (PaCO₂ >70 mmHg) is limited compared to NIMV [15]. Case series relevance: HFNC was used for post-extubation (e.g., case 2 with scoliosis) where NIMV interfaces were poorly tolerated.

In our clinical experience, we managed three cases of hypercapnic respiratory failure with varying treatment approaches. In the first case, the patient required IMV following NIMV failure due to pneumonia. *After extubation, both HFNC and NIMV were used to support a weaning combination of oxygenation and ventilatory support. HFNC improves oxygenation by providing high-flow, heated, and humidified oxygen but may not offer adequate CO₂ elimination.* NIMV (e.g., BiPAP/continuous positive airway pressure [CPAP]) increases ventilation with pressure support and corrects hypercapnia (high CO₂). Combined use may meet both needs, especially in cases of hypoxemic and hypercapnic respiratory failure. In the second case, the patient’s complex history of scoliosis, tuberculosis, and pneumonia necessitated IMV despite an initial trial with NIMV and HFNC. Post-extubation support again included HFNC and NIMV. In the third case, the patient responded well to NIMV alone without requiring escalation.

These cases illustrate the importance of individualized respiratory support strategies. While NIMV remains the cornerstone of management in hypercapnic COPD exacerbations, HFNC may serve as a supportive or bridging modality, particularly in the peri-extubation period or when NIMV is not tolerated. Although HFNC is generally less effective in severe acidosis or profound hypercapnia, it offers important comfort and secretion management advantages. Meanwhile, given its associated risks, IMV should be reserved for cases with NIMV failure or contraindications.

## Conclusions

NIV remains the preferred first-line therapy in mild-to-moderate acidosis (pH >7.25). Early intubation should be considered when severe acidosis (pH <7.25) persists or worsens. HFNC is best positioned as a post-extubation strategy or adjunct to NIV rather than a standalone treatment in severe hypercapnia. Patient-specific factors, particularly structural lung disease and comorbidities, significantly impact the success of ventilatory support.

We suggest that HFNC, NIMV, and IMV should be viewed as complementary rather than competing strategies in managing acute hypercapnic respiratory failure in COPD. HFNC may be an effective adjunct, especially in selected patients, before and after NIMV or IMV. These observations highlight the need for flexible, patient-centred approaches in respiratory care, and we hope they contribute to ongoing discussions regarding optimal ventilatory strategies in this high-risk population.
